# Benchmark Dataset and Deep Model for Monocular Camera Calibration from Single Highway Images

**DOI:** 10.3390/s25185815

**Published:** 2025-09-18

**Authors:** Wentao Zhang, Wei Jia, Wei Li

**Affiliations:** School of Mathematics and Computer Science, Shaanxi University of Technology, Hanzhong 723001, China; jiawei@snut.edu.cn (W.J.); sjliwei@snut.edu.cn (W.L.)

**Keywords:** highway scenes, multi-view surveillance, synthetic dataset, single-image calibration, deep learning

## Abstract

Single-image based camera auto-calibration holds significant value for improving perception efficiency in traffic surveillance systems. However, existing approaches face dual challenges: scarcity of real-world datasets and poor adaptability to multi-view scenarios. This paper presents a systematic solution framework. First, we constructed a large-scale synthetic dataset containing 36 highway scenarios using the CARLA 0.9.15 simulation engine, generating approximately 336,000 virtual frames with precise calibration parameters. The dataset achieves statistical consistency with real-world scenes by incorporating diverse view distributions, complex weather conditions, and varied road geometries. Second, we developed DeepCalib, a deep calibration network that explicitly models perspective projection features through the triplet attention mechanism. This network simultaneously achieves road direction vanishing point localization and camera pose estimation using only a single image. Finally, we adopted a progressive learning paradigm: robust pre-training on synthetic data establishes universal feature representations in the first stage, followed by fine-tuning on real-world datasets in the second stage to enhance practical adaptability. Experimental results indicate that DeepCalib attains an average calibration precision of 89.6%. Compared to conventional multi-stage algorithms, our method achieves a single-frame processing speed of 10 frames per second, showing robust adaptability to dynamic calibration tasks across diverse surveillance views.

## 1. Introduction

The evolution of camera calibration technology has significantly advanced video-based analysis from 2D planar to 3D spatial domains, providing critical support for 3D vision tasks such as vehicle speed calculation [1,2], spatial coordinate localization [3,4], traffic flow counting [5], and vehicle pose estimation [6,7]. This progress has substantially enhanced the environmental awareness of traffic surveillance systems. While camera calibration has established a mature theoretical framework as a fundamental computer vision technique, automatic acquisition of intrinsic and extrinsic camera parameters remains challenging in traffic surveillance scenarios due to diverse observation perspectives and unpredictable environmental conditions.

Existing automatic calibration methods can be categorized into two technical paradigms: multi-stage approaches and single-image approaches. The former achieves calibration through modularized processes including 2D-3D feature point matching, vanishing point detection, and parameter optimization, while the latter directly derives camera parameters from geometric features in single image. Classic multi-stage approaches based on the Perspective-n-Point (PnP) principle [8,9] establish mapping relationships between 3D spatial points and 2D image points to estimate camera focal length and pose parameters [10,11]. In traffic scenarios, these methods typically rely on static landmarks [12,13] or moving vehicles [14,15,16,17] to construct geometric constraint models. However, their performance is highly dependent on accurate feature point detection, making them susceptible to environmental noise such as illumination variations and shadow interference. Even minor localization errors can lead to significant deviations in calibration results.

As the most distinctive geometric feature in panoramic images, vanishing points reflect the visual convergence characteristics of camera perspective projection, with their image positions determined by both intrinsic and extrinsic camera parameters. In traffic scenes, vanishing points typically arise from two orthogonal directions: the viewpoint direction and the horizontal direction. Consequently, numerous studies have creatively utilized these two vanishing point categories for automatic camera calibration [18,19,20,21,22]. Some approaches further attempt to extract the third vanishing point from vertical objects to satisfy the Manhattan World assumption [23,24]. Nevertheless, these methods encounter challenges in highway scenarios. For instance, geometric constraints from single/dual vanishing points remain limited, necessitating supplementary prior information such as landmark dimensions, lane widths, or camera heights. The Manhattan World assumption only applies to artificial structures rather than most natural environments. Additionally, multi-stage methods involve high computational complexity due to iterative optimization across modules. Particularly for Pan-Tilt-Zoom (PTZ) monitoring cameras, continuous detection of lane markings or vehicle targets to stabilize vanishing point acquisition often prematurely terminates calibration procedures during focal length/pose adjustments.

Therefore, developing efficient and robust fully automatic camera calibration methods holds significant practical value. Based on geometric principles of camera imaging, image perspective features provide critical constraints for solving camera parameters. Compared with traditional algorithms relying on scene priors, deep learning frameworks demonstrate stronger environmental adaptability through data-driven feature extraction mechanisms. Previous studies have demonstrated that convolutional neural networks (CNNs) can localize vanishing points. The work [25] directly regressed vanishing point coordinates from panoramic images. Another category of approaches [26,27] reformulates the vanishing point detection task as a classification problem by discretizing the image space into *n* × *n* grids, then using the softmax classifier to predict grid positions containing vanishing points. Similarly, a vanishing point representation method [28] based on quadrant partitioning offers new insights for camera parameter estimation. In recent years, deep learning frameworks have extended to end-to-end single-image calibration through supervised learning, directly regressing camera focal lengths and other parameters [29]. The core motivation of these methods stems from utilizing observable visual cues in images, such as horizon features [30,31,32] and scene vector fields [33]. However, in highway scenarios, such visual cues are often weakened due to homogeneous road structures and diverse camera viewpoints, significantly degrading performance of existing methods. More critically, the scarcity of publicly available highway scene datasets remains a persistent challenge, leaving camera calibration research as an unresolved problem.

To address these challenges, this paper proposes an automatic calibration framework for highway surveillance cameras using a single image, featuring three primary contributions. (1) We constructed a large-scale synthetic dataset using the CARLA [34] simulation engine, containing 6 map categories and 36 representative highway segments. Through automated annotation pipelines, we generated 336,249 images with ground-truth calibration parameters. This dataset closely matches real-world highway scenarios in camera perspectives, road geometries, and weather conditions, significantly reducing deep learning models’ reliance on real-world data. (2) We developed a deep calibration network (DeepCalib) that synergistically integrates the triplet attention module (TAM) [35]. This architecture enhances semantic representation of perspective projection features, enabling joint estimation of vanishing point coordinates and camera pose parameters from single images while automatically adapting to varying observation viewpoints. (3) We adopted a dual-stage training paradigm combining synthetic pre-training and real-data fine-tuning. Robust feature learning is first performed on synthetic data with augmentation strategies to improve generalization. Subsequent parameter fine-tuning on limited real-world data enables virtual-to-real transfer learning. Experimental results demonstrate this approach significantly enhances model adaptability in complex traffic environments.

The rest of this paper is organized as follows. Section 2 introduces the proposed synthetic dataset. Section 3 details the calibration model, network architecture, and training methodology. Section 4 presents experimental results including comprehensive comparisons with baseline models. Finally, Section 5 concludes the study and explores future research directions.

## 2. Benchmark Dataset

Large-scale annotated datasets play a pivotal role in enhancing the generalization capability of deep learning models for visual perception tasks. However, existing public highway-scene datasets predominantly exhibit single-view limitations and lack complete annotations of camera intrinsic and extrinsic parameters, which hinders their capacity to support the training demands of high-precision visual perception models. While the prior work [3] has released Multi-View Camera Calibration Dataset (MVCCD), the sample sizes remain insufficient to cover the diversity of complex highway scenarios. To address this gap, we constructed a large-scale synthetic dataset using the CARLA [34] traffic simulation platform, employing virtual scene augmentation strategies to explicitly expand data distribution diversity.

**Figure 1:**
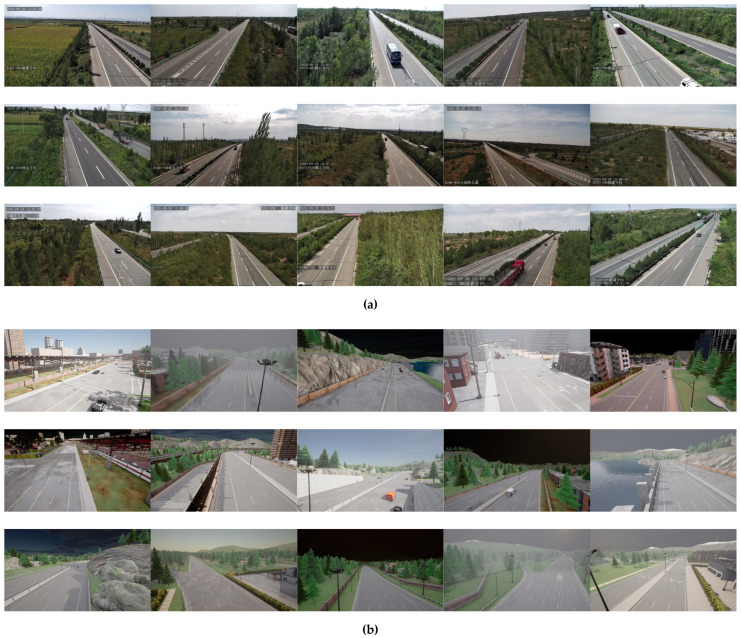
Representative images of different scenarios in real dataset and synthetic dataset. (**a**) Real dataset; (**b**) Synthetic dataset.

We selected 36 arterial roads from 6 virtual city maps as foundational scenarios. Each road randomly deployed three camera groups (left/center/right) to achieve multi-view coverage. A comprehensive weather simulation system was developed using procedural generation for typical meteorological conditions including sunny, rainy, cloudy, foggy, and nighttime scenarios, ensuring deep networks maintain robust performance under diverse weather patterns. The traffic flow simulation module incorporated 33 standardized vehicle models with dynamic adjustment capabilities ranging from sparse to dense traffic conditions, maintaining consistency with real highway vehicle density parameters. To simulate operational boundaries of traffic surveillance cameras, we defined four parameter sampling spaces: field-of-view (FOV) [70°, 120°], pitch angle [−28°, 0°], yaw angle [−40°, 40°], and mounting height [10 m, 14.5 m]. Random parameter sampling ensures uniform label distribution across image regions, effectively mitigating training biases caused by imbalanced datasets. Figure 1 illustrates representative synthetic scenes that closely resemble real-world highway environments while exhibiting greater diversity in camera viewpoints and road geometries.

The final dataset comprises 336,249 pairs of 1920 × 1080 resolution RGB images with corresponding annotations. Each annotation file records vanishing point coordinates, pitch angle (ϕ), yaw angle (θ), camera focal length (*f*), and camera height (*h*). Data partitioning follows a stratified sampling strategy, allocating samples to training/validation/test sets in 7.5:1.5:1 ratios. Table 1 compares parameter distributions between the real-world dataset (MVCCD_R) and synthetic counterpart (MVCCD_S), demonstrating broader coverage across all dimensions for the proposed dataset.

**Figure 2:**
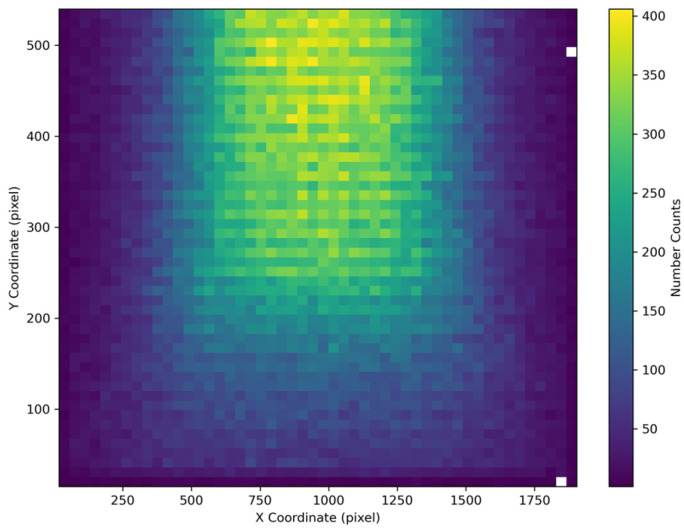
Distribution of vanishing point coordinates in MVCCD_S.

We systematically demonstrate the parameter distribution characteristics of the constructed dataset by visualizing histograms of vanishing point coordinates, camera focal lengths, and pose parameters. Figure 2 reveals that vanishing point coordinates cover the majority of the image plane. Notably, due to the typical top-down installation of surveillance cameras, vanishing points exhibit a pronounced bias toward the upper image half. This distribution pattern closely aligns with the visual perception of roads receding into the distance in real-world scenarios.

**Figure 3:**
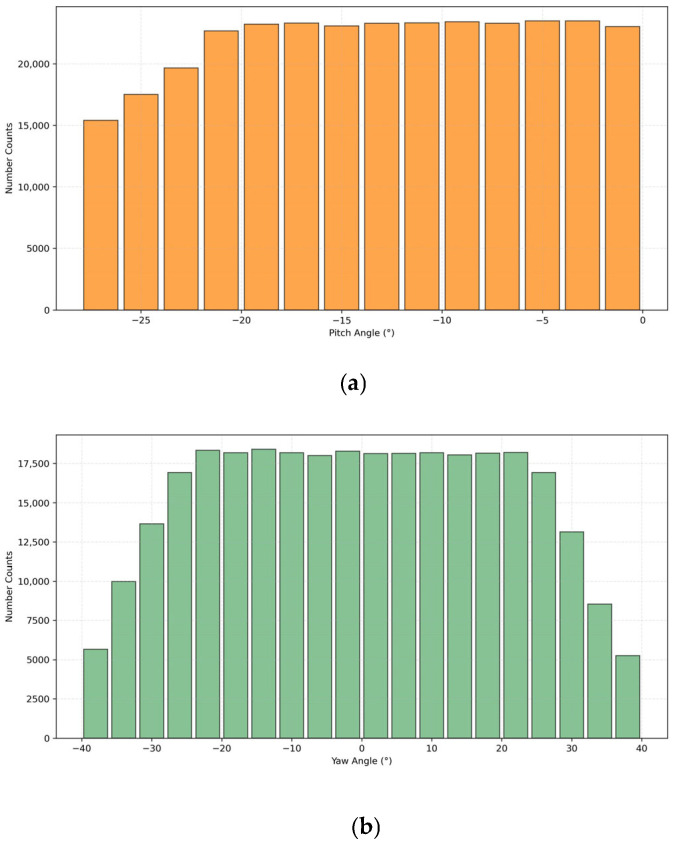

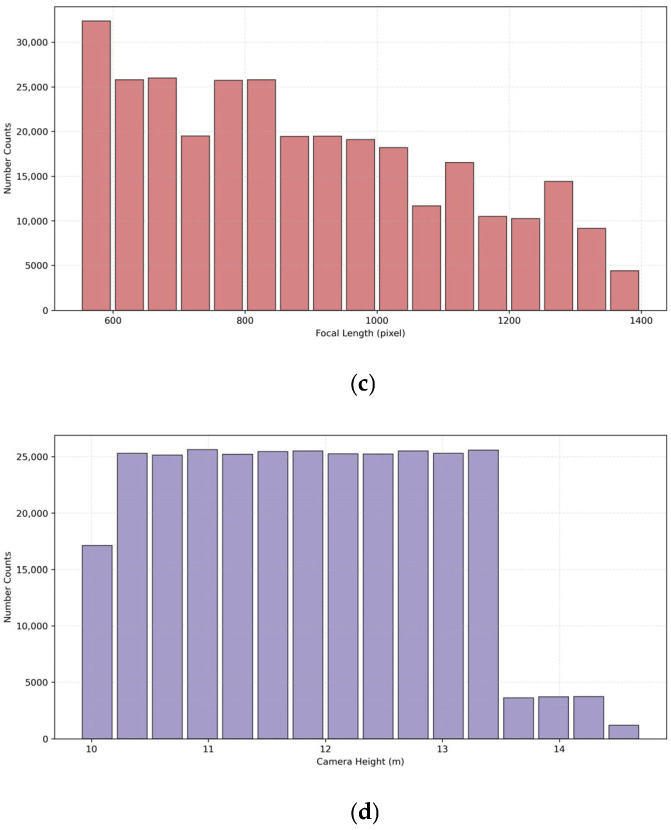
Histograms of camera parameters in MVCCD_S. (**a**,**b**) represent the distributions of pitch angle and yaw angle respectively, while (**c**,**d**) correspond to the distributions of focal length and camera height respectively.

Figure 3 presents statistical histograms of camera parameters in MVCCD_S. The two rotation angles (pitch/yaw) exhibit uniform distributions across their defined angular spaces. The focal lengths demonstrate a broad distribution across 500–1400 pixels, with equivalent focal lengths spanning the operational spectrum from wide-angle to medium-telephoto configurations typical of surveillance systems. Camera height parameters cluster within 10.0–13.5 m, aligning with empirical deployment standards. The dataset maintains statistical equilibrium across critical parameters, providing an ideal benchmark for validating camera calibration algorithms based on geometric constraints.

**Figure 4:**
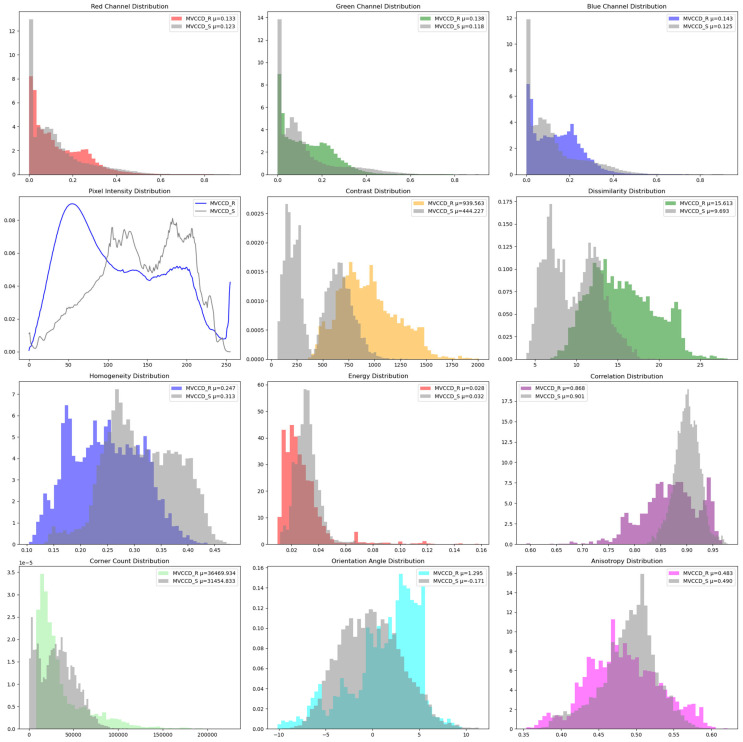
Quantitative comparison between MVCCD_S and MVCCD_R datasets across multiple feature dimensions.

Figure 4 presents a quantitative comparison between MVCCD_S and MVCCD_R datasets across multiple feature dimensions, including RGB color channels, texture features, pixel intensity, and geometric properties. Through visualized histograms and statistical mean overlays, the following conclusions are drawn:

**Color Space Distribution**: Synthetic data exhibits slightly lower RGB channel means compared to real data, indicating overall darker brightness. This observation is directly attributed to simulated weather conditions (rain, fog, nighttime) in synthetic scenes, which shift pixel values toward lower luminance regions.

**Texture Complexity**: Real data demonstrates significantly higher contrast, suggesting richer edge details and high-frequency textures. The disparity in dissimilarity and homogeneity further confirms the regularity of synthetic textures—exhibiting stronger spatial correlation—while real data shows lower texture homogeneity due to natural noise and structural complexity.

**Pixel Intensity Dynamics**: Real data intensity concentrates in the 10–240 range with a left-skewed peak at 50 gray levels. Synthetic data spans the 20–250 range with bimodal peaks (115 and 180), demonstrating enhanced diversity through simulations of varying weather (sunny/cloudy) and time periods (day/dusk).

**Geometric Feature Consistency**: Close alignment in orientation angle and anisotropy indicates high statistical consistency between datasets in object orientation and shape anisotropy. The corner count discrepancy suggests room for improvement in modeling complex geometric details, but synthetic data’s directional distribution adequately covers real-world variations.

Overall, synthetic and real datasets demonstrate significant statistical consistency in geometric features, particularly in orientation angle and anisotropy metrics. Color and texture discrepancies highlight the necessity of data augmentation techniques such as stochastic color jittering and noise injection to further improve distributional alignment between synthetic and real-world scenes.

## 3. Methods

### 3.1. Calibration Model for Traffic Surveillance Cameras

Calibration methods for traffic surveillance cameras have been extensively discussed, with detailed derivations referenced in the work [36]. This section provides a concise overview of the underlying principles. In the standard calibration model, a homogeneous 3D spatial point ***P*** = [*X*,*Y*,*Z*,1]^T^ is projected onto the image plane as a 2D point ***p*** = [*u*,*v*,1]^T^ through the projection matrix M.

(1) $$\lambda {\left[u,v,1\right]}^{T}=M{\left[X,Y,Z,1\right]}^{T},$$

The general mathematical formulation of ***M*** is expressed as ***M*** = ***K***[***R*|*T***], where ***K*** denotes the camera’s intrinsic parameters (including focal length and principal point coordinates), while ***R*** and ***T*** represent the extrinsic parameters (relative to the world coordinate system), corresponding to the rotation matrix and translation vector, respectively.

**Figure 5:**
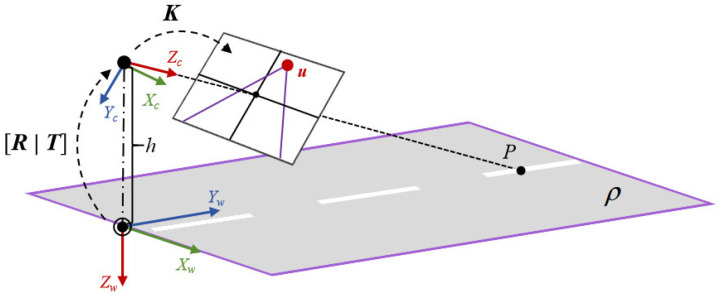
Traffic surveillance camera calibration model. The camera is placed at a height of h meters above the ground plane. *X_w_*-*Y_w_*-*Z_w_* axis represents the world coordinate system, while *X_c_*-*Y_c_*-*Z_c_* axis represents the camera coordinate system. U denotes the vanishing point along the road direction. ρ indicates the ground plane.

For traffic surveillance cameras, the calibration parameters can be simplified by establishing a rational world coordinate system (refer to Figure 5). Furthermore, under the assumptions that the camera’s principal point coincides with the image center and the roll angle remains zero, the projection matrix ***M*** is solely determined by the focal length *f*, pitch angle ϕ, yaw angle θ, and camera height *h*.

As a fundamental characteristic of perspective projection, vanishing points exhibit strong correlations with the camera’s focal length, pitch angle, and yaw angle. Their coordinates (*u*,*v*) in the image plane can be derived using the following relationships:

(2) $$\left\{\begin{array}{c} u\;=\;c_{x}+f\cdot \frac{tan\theta }{cos\phi }\\ v\;=\;c_{y}+f\cdot tan\phi \end{array}\right.,$$

where (*c_x_*,*c_y_*) denotes the coordinates of the principal point. As demonstrated in Equation (2), the focal length *f* can be derived given the vanishing point coordinates (*u*,*v*), pitch angle ϕ, and yaw angle θ. When combined with the camera height *h*, these parameters enable the complete construction of the calibration matrix.

### 3.2. Single-Image Calibration with DeepCalib

**Figure 6:**
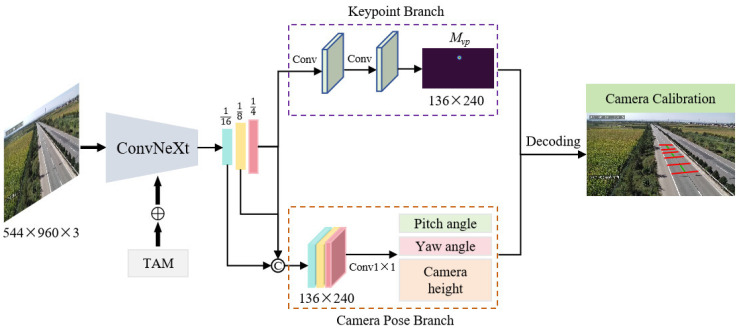
The overall framework of DeepCalib. TAM stands for the triplet attention module. The symbol ⊕ denotes feature fusion and © indicates feature map connection. The keypoint branch outputs vanishing point heatmap, and the camera pose branch estimates extrinsic parameters.

Three-dimensional objects exhibit distinct visual convergence effects after undergoing camera perspective projection transformations. In traffic scenes, geometric deformations and convergence directions of road structures vary significantly across different viewpoints, with these projection patterns universally present in both panoramic images and local objects. This implies that camera parameters can be derived from image projection features. According to this geometric regularity, we developed DeepCalib, a single-image based deep calibration network whose overall framework is illustrated in Figure 6.

The DeepCalib architecture comprises three components: a backbone, a deconvolutional module and a multi-task detection head. The backbone network, built on the ConvNeXt [37] architecture, integrates the TAM [35] module for cross-dimensional feature fusion. After feature encoding, three-stage deconvolutional modules perform progressive upsampling, ultimately generating multi-scale feature maps at 1/16, 1/8, and 1/4 resolutions of the original image. The multi-task detection head contains a key point localization branch and a camera pose estimation branch to perform geometric inference from the captured features. Based on the established calibration model, the network outputs are decoded to obtain both intrinsic parameters (focal length) and extrinsic parameters (rotation angles, translation vectors).

#### 3.2.1. Backbone

The backbone network adopts a ConvNeXt architecture to jointly capture global and local visual features. Its hierarchical design incorporates four cascaded ConvNeXt Block modules, constructing multi-scale feature representations through progressive down sampling and channel expansion. For feature extraction, each ConvNeXt Block replaces 3 × 3 convolutions with 7 × 7 kernels, maintaining local texture modeling capability while expanding receptive fields to capture long-range spatial dependencies. This design enables joint encoding of global semantic contexts and fine-grained local patterns through enhanced feature hierarchies.

In convolutional neural networks, attention mechanisms enable the model to focus on specific visual regions or assign differentiated weights to different regions, thereby filtering critical features from vast information. A typical example is SENet [38], which captures inter-channel importance differences through channel attention. However, its lack of spatial dimension perception leads to insufficient modeling of spatial positional correlations. Although Convolutional Block Attention Module (CBAM) [39] integrates channel and spatial attention, it fails to establish cross-dimensional feature interaction mechanisms. Given the pervasive perspective projection characteristics in panoramic images and local objects, accurately capturing global-local features and their interactions is pivotal for enhancing network performance. The TAM module effectively addresses spatial-channel dimensional feature interactions through three parallel branches. Each branch independently aggregates interaction information between specific dimensions and channel dimensions in the input, forming a cross-dimensional information enhancement mechanism.

**Figure 7:**
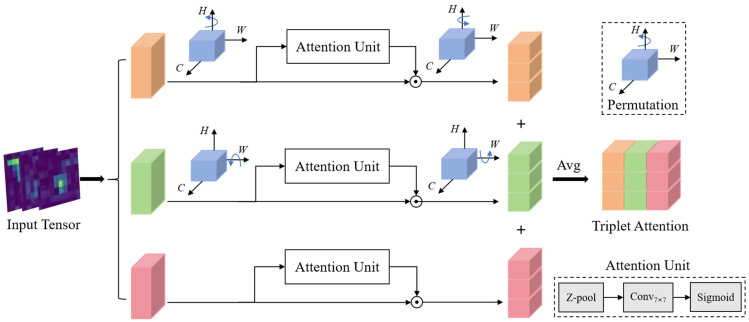
The overall framework of TAM [35].

To this end, this paper integrates the TAM module into the backbone network, establishing a joint modeling framework for channel-spatial dimensional dependencies. Specifically, we embed a TAM unit within each ConvNeXt module to synchronously extract low-level geometric features and high-level semantic features. As illustrated in Figure 7, the TAM module achieves interaction among channel height (CH), channel width (CW), and spatial attention (HW) through three parallel branches. Each branch follows a three-stage processing pipeline of “Z-pool operation—convolution—Sigmoid activation”, and ultimately generates an attention-weighted tensor of the same dimension as the original feature through point-wise multiplication with the original feature. Specifically, the Z-pool layer compresses the zeroth dimension of the tensor to two dimensions by concatenating the features obtained from average pooling and maximum pooling across that dimension. Mathematically, it can be formalized as follows:

(3) $$Z-pool(\chi )=[{MaxPool}_{0d}(\chi ),{AvgPool}_{0d}(\chi )],$$

where 0*d* is the 0th-dimension across which the max and average pooling operations take place.

The first branch is responsible for constructing the interaction relationship between channels and height. For an input tensor $\chi \in R^{C\times H\times W}$, it is first rotated 90° anti-clockwise along the *H*-axis to form $\chi_{1}\in R^{W\times H\times C}$. Subsequently, $\chi_{1}$ is compressed to a dimension of 2 × *H* × *C* via Z-pool, and then passed through a convolutional layer and a batch normalization layer to generate attention weights. These weights are activated by a Sigmoid function (σ) and applied to $\chi_{1}$. After that, it is rotated 90° clockwise along the *H*-axis again to restore the same shape as the original input tensor χ. This branch utilizes height-dimension information to focus on the vertical geometric features of the image, enabling the estimation of the vertical vanishing point (*v*) and the pitch angle (ϕ). The operation process can be formally described as follows:

(4) $$\omega_{CH}=\sigma ({Conv}_{7\times 7}(Z-pool(\chi_{1})))⊙\chi_{1},$$

where ⊙ denotes broadcast element wise multiplication.

The second branch deals with channel-width interaction. Similarly, the input χ is rotated 90° anti-clockwise along the *W*-axis to generate $\chi_{2}\in R^{H\times C\times W}$. Subsequently, a three-stage processing operation is employed to generate channel-width attention. This branch estimates the horizontal vanishing point (*u*) and the camera yaw angle (θ) by perceiving features in the horizontal dimension. The formulation is presented as follows:

(5) $$\omega_{CW}=\sigma ({Conv}_{7\times 7}(Z-pool(\chi_{2})))⊙\chi_{2},$$

The third branch directly processes the spatial dimension. The input χ is compressed by Z-pool to a 2 × *H* × *W* dimension. The simplified tensor $\chi_{3}$ captures global contextual dependencies through a 7 × 7 convolution. After sigmoid activation, it generates 1 × *H* × *W* attention weights that directly act on the original input χ. This branch enhances the overall perception of road geometry and camera perspective through spatial dimension modeling.

(6) $$\omega_{HW}=\sigma ({Conv}_{7\times 7}(\chi_{3}))⊙\chi$$

Finally, the *C* × *H* × *W* dimensional fine-tuned attention weights generated by the three branches are fused across dimensions through simple averaging, with the aggregation process expressed as:

(7) $$\hat{\chi }=\frac{1}{3}{(\omega }_{CH}+\omega_{CW}+\omega_{HW})$$

This architecture preserves the integrity of the original feature space structure while achieving cross-dimensional synergistic enhancement of channel-spatial features. It enables the network to adaptively focus on key perspective-sensitive feature regions, significantly improving the accuracy of vanishing point detection and the robustness of camera rotation angle estimation. These capabilities provide strong support for geometric structure perception in real-world scenarios.

#### 3.2.2. Multi-Task Detection Head

The multi-task detection head adopts a dual-branch architecture comprising a keypoint branch and a camera pose branch, responsible for vanishing point detection and camera pose estimation. The keypoint branch treats the vanishing point along road extension directions as a critical geometric anchor in panoramic imagery. This branch processes 1/4-scale feature maps from the upsampling module, employing two cascaded 1 × 1 convolutional layers for channel dimension reduction, ultimately generating heatmap at 136 × 240 resolution. During ground truth generation, a 2D Gaussian kernel was used to construct the vanishing point response region, with peak coordinates corresponding to the true vanishing point location. Sub-pixel localization accuracy is achieved through heatmap peak response decoding, enabling precise geometric anchor localization in complex traffic environments.

**Figure 8:**
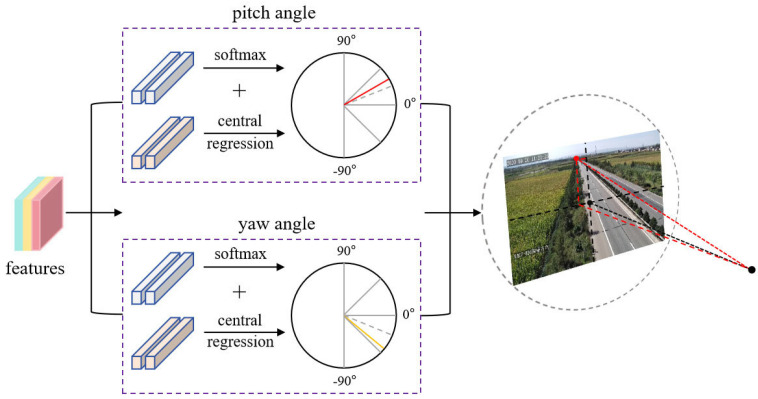
Schematic diagram of rotation angle estimation.

For rotation angle estimation, direct regression of continuous angular values is prone to prediction instability. Inspired by the MultiBin [40] architecture, the camera pose estimation branch adopts a classification and central-residual regression strategy. As illustrated in Figure 8, the rotation angle space is discretized into *n* overlapping bins. The network first predicts a probability distribution over these bins, then performs residual ($\delta_{\eta }$) regression relative to the selected bin’s central angle. The final rotation calibration is obtained through summation of the bin center value and predicted residual.

(8) $$\hat{\eta }=c_{i}+\delta_{\eta_{i}},$$

where $\hat{\eta }$ represents the ground truth, $c_{i}$ denotes the center angle of the bin i and $\delta_{\eta_{i}}$ refers to the residual with respect to the center of the bin i.

Regarding camera height regression, a hybrid strategy combining global prior and local refinement was employed. We precomputed a mean height ($\bar{h}$) across the entire dataset, with the network only required to predict residual offset ($\delta_{h}$) relative to this global prior. This approach significantly reduces parameter search space complexity while maintaining adaptive calibration capability. The absolute camera height for each input image is recomputed by combining the global mean value with the predicted residual offset.

(9) $$h=\bar{h}\cdot e^{\delta_{h}},$$

where the height residual $\delta_{h}$ is activated using the sigmoid function σ. $\delta_{h}=\sigma (o_{h})-1/2$. *o* stands for the specific output of the network.

#### 3.2.3. Multi-Task Loss Function

Based on the network outputs, the loss function of DeepCalib comprises four components: vanishing point classification loss $L_{v}$, offset loss $L_{vo}$, multibin loss $L_{m}$, and camera height residual loss $L_{ho}$. The vanishing point loss is computed using focal loss [41]:

(10) $$L_{v}=-\frac{1}{N}\sum_{i=1}^{H}\sum_{j=1}^{W}\left\{\begin{array}{c} {\left(1-{\hat{P}}_{c_{ij}}\right)}^{\alpha }log({\hat{P}}_{c_{ij}}),\;\;\;\;\;\;\;\;{\;\;\;\;\;\;P}_{c_{ij}}=1\;\;\;\;\;\;\;\;\;\\ {\left(1-P_{c_{ij}}\right)}^{\beta }{\hat{p}}_{c_{ij}}^{\alpha }log({1-\hat{P}}_{c_{ij}}),\;\;\;\;\mathrm{otherwise} \end{array}\right.,$$

where (*H*,*W*) denotes the heatmap size, and *N* represents the number of positive samples. The terms $P_{c_{ij}}$ and ${\hat{P}}_{c_{ij}}$ correspond to the ground truth and predicted responses at heatmap position (*i*,*j*), respectively. The hyperparameters α and β adjust the loss weights for positive and negative samples, respectively. To compensate for the quantization error due to feature map down sampling, the vanishing point offset loss is calculated as follows:

(11) $$L_{vo}=\frac{1}{N}\left|\delta_{p}-(\frac{P}{R}-\tilde{P})\right|,$$

where *P* represents the actual vanishing point coordinates, *R* denotes the down sampling factor. $\delta_{p}$ is the true offset, while $\tilde{P}=\left\lfloor \frac{P}{R}\right\rfloor$ (the symbol $\left\lfloor \cdot \right\rfloor$ indicates the floor operation).

(12) $$L_{m}=L_{c}{+\omega \times L}_{co},$$

where ω is the weighting factor, set to 0.5. The confidence loss $L_{c}$ is described by the softmax loss for each bin, while $L_{co}$ aims at eliminating the discrepancy between the predicted and true values within each bin. The calculation formula is as follows:

(13) $$L_{co}=-\frac{1}{m}\sum cos(\hat{\eta }-c_{i}-\delta_{\eta_{i}}),$$

where *m* denotes the number of bins covering the true angle, and $\hat{\eta }$ represents the ground truth angles. For the camera height residual loss $L_{ho}$, each regression quantity is evaluated using the Smooth L1 loss:

(14) $$L_{ho}=\sum Smooth\;L1(\delta_{h}),$$

In summary, the total loss function Loss of DeepCalib can be described as follows:

(15) $$Loss=\omega_{1}\times L_{v}+\omega_{2}\times L_{vo}+\omega_{3}\times L_{m}+\omega_{4}\times L_{ho},$$

where $\omega_{1}$, $\omega_{2}$, $\omega_{3}$, and $\omega_{4}$ are the weighting factors between the sub–loss functions, with $\omega_{1}+\omega_{2}+\omega_{3}+\omega_{4}=1$.

### 3.3. Training Details

This section presents a two-stage progressive training paradigm: (1) end-to-end robust feature learning on MVCCD_S without pre-trained weight initialization, followed by (2) task-specific parameter fine-tuning of the multi-task detection head on MVCCD_R. The initial training phase involves the implementation of several data processing procedures.

**Preprocessing**: During synthetic data generation, viewpoint diversity was simulated through random perturbations of camera parameters. Despite constrained parameter perturbation ranges, certain samples exhibit vanishing points near image boundaries, causing their corresponding heatmap responses to exceed valid perceptual ranges after down sampling. To address this, a data purification step was first implemented to exclude such invalid samples. The retained valid image sequences were then resized to 544 × 960 pixels as standardized input for supervised training.

**Data Augmentation**: The training pipeline incorporated three data augmentation techniques: horizontal flipping, spatial translation, and color transformation. Horizontal flipping and random translation were applied with a probability of 0.4. Translation vectors were randomly combined from four directions (up/down/left/right), with two safety mechanisms: (1) a 50-pixel displacement threshold per direction, aborting transformations exceeding this limit, and (2) a maximum translation magnitude of 180 pixels. Void regions generated post-translation are filled using nearest-neighbor interpolation to maintain pixel continuity. During horizontal flipping, simultaneous sign inversion of the yaw angle ensures parameter validity. The color augmentation module includes random jittering of brightness/contrast/saturation and Gaussian noise injection. To prevent over-enhancement, this operation was activated with a probability of 0.2.

**Hyperparameter Choice**: Vanishing point heatmaps were generated using 2D Gaussian masks with radius *r* = 8, where pixels with mask values ≥ 0.5 were defined as positive samples. For heatmap loss calculation, parameters were configured as α=2 and β=4. For angular discretization, pitch angle ϕ and yaw angle θ were partitioned using 3 and 5 overlapping bins, respectively. The specific binning parameters were configured as: 12° width with 4° overlap for pitch angles, and 20° width with 5° overlap for yaw angles. Pre-computed statistical analysis yielded average camera height of 11.83 m for MVCCD_S and 12.36 m for MVCCD_R.

**Training Strategy**: The training process adopted a two-stage paradigm combining pre-training and fine-tuning. During the pre-training phase, comprehensive feature learning was conducted on large-scale synthetic datasets using a batch size of 32 across 20 epochs (157,600 iterations) with an initial learning rate of 2 × 10^−3^. This stage emphasizes robust representation learning through end-to-end optimization of all network parameters. The subsequent fine-tuning stage focused on task-specific parameter refinement for the multi-task detection head using real-world datasets. This phase employed a reduced batch size of 16 over 10 epochs (6720 iterations) with an adjusted initial learning rate of 2 × 10^−5^. Both training stages utilized the AdamW [42] optimizer with weight decay regularization and implement dynamic learning rate adjustment: the learning rate was decayed by a factor of 0.1 when validation loss shows no improvement for three consecutive epochs. To accelerate convergence, distributed training was performed across four NVIDIA A800 GPUs using data parallelism.

## 4. Experiments

To validate the proposed method, we conducted five experiments: (1) ablation studies, (2) binning Strategy, (3) transfer learning, (4) camera calibration, and (5) time consumption. All experiments were performed under identical conditions using a unified test set (comprising both MVCCD_S and MVCCD_R datasets), consistent hardware configuration (NVIDIA A800 GPU) and hyperparameter settings to ensure direct comparability of results.

### 4.1. Ablation Studies

This study employed three ConvNeXt sub-architectures (ConvNeXt_tiny, ConvNeXt_small, and ConvNeXt_base) as baseline models. Enhanced networks were developed by integrating TAM modules into these backbones. Ablation experiments were systematically conducted to compare performance differences between baseline and enhanced models. All evaluations were performed on MVCCD_S, with quantitative analysis focusing on four key metrics: vanishing point Euclidean distance (L2 Dis), the Mean Absolute Error (MAE) of pitch angle, yaw angle, and camera height. Experimental results are summarized in Table 2.

Statistical analysis reveals a nonlinear positive correlation between model capacity and performance in the ConvNeXt series. As network scale increased from tiny to base, continuous optimization is observed across all evaluated metrics. Notably, the transition from ConvNeXt_tiny to ConvNeXt_small yields the most significant performance improvements: L2 distance error decreases by 11.17 pixels, while pitch and yaw angle errors reduce by 3.97° and 4.87°, respectively. However, when model capacity is further expanded to the base level, the rate of performance improvement markedly slowed, with only a 5.72-pixel reduction in L2 distance error and 0.99° and 1.68° reductions in pitch and yaw angle errors, respectively. This phenomenon indicates a feature representation bottleneck in lightweight models, while mere capacity scaling fails to deliver sustained linear performance gains.

Ablation studies demonstrate that integrating TAM into all baseline models yields significant improvements across four core metrics. Specifically, vanishing point localization errors are reduced by 16.32–22.46 pixels, with ConvNeXt_base_TAM achieving the optimal 22.22-pixel reduction. Pitch/yaw errors for ConvNeXt_base_TAM decrease to 1.33° and 2.45°, representing reductions of 0.41° and 1.49° (23.56% and 37.82% decreases) compared to the baseline model. These results confirm that the TAM module effectively enhances baseline models’ perception of scene geometric structures. While height residual estimation shows relatively modest improvements, the enhanced model maintained stable performance with a mean absolute error (MAE) of 0.89 m versus the baseline’s 0.91 m. This limited enhancement may stem from inherent properties of perspective projection features—camera height does not directly influence perspective effects, resulting in reduced model sensitivity to height variations.

### 4.2. Binning Strategy

Based on results in Section 4.1, ConvNeXt_base_TAM was selected as the test network to systematically validate rotation angle binning strategies. In these experiments, pitch and yaw angles were partitioned into 2–6 bins with 4° and 5° overlaps, respectively. Classification accuracy and MAE of residual angles served as primary evaluation metrics. Table 3 reveals a distinct trade-off between classification and regression performance as bin counts increased from 2 to 6. For pitch angle: At 2 bins, classification accuracy peaked at 94.23% but with substantial regression error (2.78°). When bin count reached 6, accuracy declined to 79.03% while MAE improved to 3.35°. Similarly, yaw angle showed highest classification accuracy at 2 bins (92.37%) with correspondingly high regression error (5.27°), but accuracy dropped to 76.91% and MAE reduced to 4.45° at 6 bins. This inverse relationship suggests that fewer bins enhance classification discriminability but expand regression range, increasing error. Conversely, more bins refine angular resolution to reduce regression error but blur classification boundaries, compromising accuracy.

### 4.3. Transfer Learning

This section presents experimental validation of the transfer learning strategy applied to the DeepCalib model. The implementation involved two training paradigms: (1) pre-training from scratch on MVCCD_S followed by fine-tuning on MVCCD_R, and (2) direct training exclusively on MVCCD_R as a baseline comparison. Given the differences in RGB color channels and texture feature distributions between synthetic and real images, this experiment first employed data augmentation techniques such as random color jittering and noise injection to optimize the synthetic dataset, thereby mitigating distribution bias between the two datasets.

**Figure 9:**
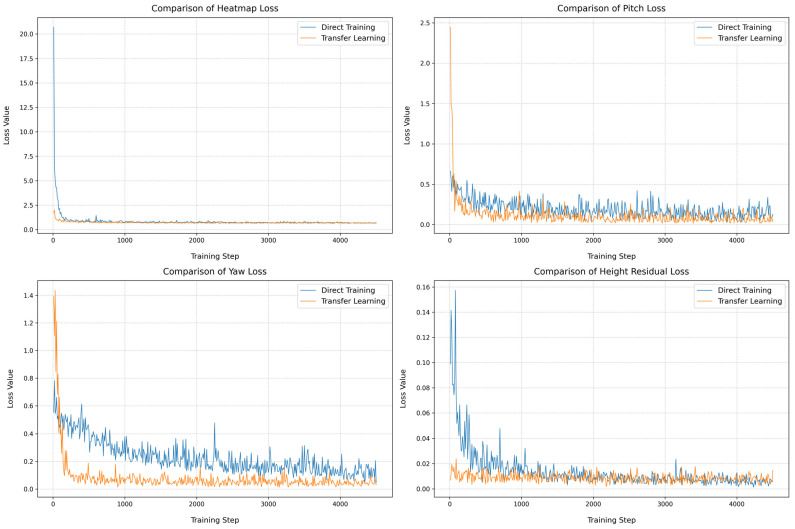
Loss curves for direct training and transfer learning.

Figure 9 systematically illustrates the evolution of loss functions under both strategies, including vanishing point heatmap loss, pitch angle estimation loss, yaw angle estimation loss, and camera height residual loss. Global analysis reveals that compared to direct training, the transfer learning model achieves consistently lower loss values across all metrics, with significantly reduced fluctuations in loss curves during training. Although the initial loss for rotation angle training is higher in the transfer learning approach, its curves demonstrate faster convergence. This indicates that pre-training on synthetic data effectively enhances generalization capability.

**Figure 10:**
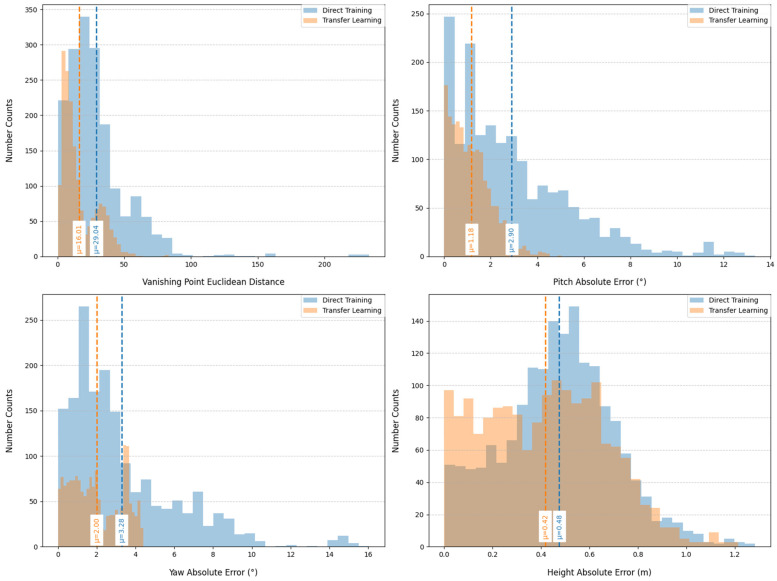
Quantitative analysis of direct training and transfer learning.

Figure 10 provides quantitative comparisons on MVCCD_R. For vanishing point detection, the transfer learning strategy constrains Euclidean distance errors within the [0, 50] pixel range (mean 16 pixels), significantly outperforming direct training’s [0, 220] pixel range (mean 29 pixels). In camera extrinsic parameter estimation, absolute errors for pitch and yaw angles are confined to [0°, 4°] (mean 1.18°) and [0°, 4.2°] (mean 2°), respectively, substantially surpassing direct training results of [0°, 13°] (mean 2.90°) and [0°, 16°] (mean 3.28°). Camera height residuals are controlled within 1.2 m (mean 0.42 m), improving upon direct training’s 0.48 m average. All quantitative metrics confirm the superior performance of transfer learning across evaluated dimensions.

**Figure 11:**
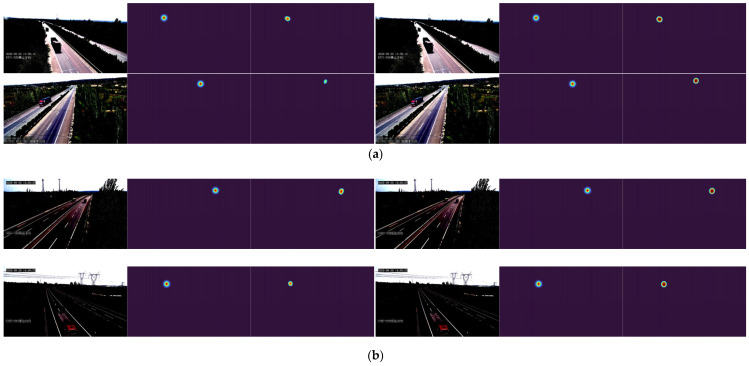
Qualitative comparison of vanishing point detection performance by DeepCalib on real-world test images. (**a**) Straight road scenarios; (**b**) Curved road scenarios. For each row, the left image shows results from a model trained exclusively on MVCCD_R, while the right image demonstrates outcomes after pre-training on MVCCD_S followed by fine-tuning on MVCCD_R.

To intuitively verify transfer learning efficacy, Figure 11 compares qualitative results of both strategies in real-world vanishing point estimation. The experimental setup comprises four typical road scene groups arranged in side-by-side format: left panels show direct training predictions, while right panels display transfer learning results. Columns sequentially present input images, ground truth heatmaps, and predicted heatmaps. Observations indicate that transfer learning produces consistently stable vanishing point detection across diverse road environments, particularly excelling in complex curved scenarios with heatmap distributions showing greater consistency with ground truth.

These qualitative findings align with quantitative results, conclusively demonstrating that the proposed synthetic dataset serves as a valuable complement to real-world data, enabling significant performance improvements in practical applications through transfer learning. The statistical consistency of geometric features plays a positive role in enabling DeepCalib to learn the geometric structure and camera view properties of real-world scenes. Although the introduction of weather conditions such as rain, fog, and night in the synthetic dataset results in slightly lower RGB color channel values compared to real scenes, and the inherent homogeneity of images caused by virtual engine characteristics remains an issue, the implementation of reasonable data augmentation strategies (including random color jittering and noise injection) effectively reduces dataset distribution discrepancies. This approach significantly improves the performance of vanishing point detection accuracy and camera rotation angle estimation in highway scenarios when applying transfer learning to real-world applications.

### 4.4. Camera Calibration

This section comprehensively evaluates DeepCalib’s calibration performance in real-world road scenarios through two experiments. As a foundational validation, we assessed DeepCalib’s vanishing point estimation capability on MVCCD_R and another public dataset [27], performing comparative analysis against representative traditional methods (AutoCalib [19] and Edgelets [43]) and state-of-the-art deep learning approaches [3,27]. Considering that AutoCalib and Edgelets apply exclusively to video sequences with fixed camera views, we specifically used videos originating from the same scenarios as MVCCD_R to ensure fair comparison. Identical configurations were adopted for subsequent related experiments. To address resolution discrepancies between datasets, this study employed L2 and normalized distance (NormDis) for quantitative evaluation. The NormDis, standardized by image diagonal length, effectively eliminated resolution variations’ impact on assessment results, enabling cross-dataset objective comparison. The formulation is presented as follows:

(16) $$NormDis=\frac{\left\|\hat{vp}-vp\right\|}{\ell_{d}},$$

where $\hat{vp}$ denotes the estimated vanishing point coordinates, while *vp* represents the ground truth vanishing point coordinates, and $\ell_{d}$ indicates the image diagonal length.

Table 4 systematically presents comparative results of the five methods. On the MVCCD_R dataset, DeepCalib achieved L2 distance errors of 13 pixels for straight roads and 34 pixels for curved roads, outperforming other algorithms by 44–78 pixels and 39–96 pixels, respectively. On dataset [27], it reduces L2 errors by 2 pixels and 7 pixels compared to DeepCN and DeepVP. NormDis metrics shows DeepCalib attains 0.006 (straight roads) and 0.022 (curved roads) on MVCCD_R, and 0.014 on dataset [27], all significantly lower than traditional methods (e.g., 0.059 for Edgelets on curved roads) and deep learning approaches (e.g., 0.026 for DeepCN on straight roads). This confirms that DeepCalib’s vanishing point localization accuracy is resolution-independent, depending only on scene complexity, which substantially enhances stability in cross-device and cross-scene deployments.

Notably, curved road scenarios exhibit significantly higher vanishing point estimation errors than straight roads. This discrepancy arises because nonlinear road edge distributions challenge traditional methods reliant on linear assumptions. Meanwhile, deep learning approaches also suffer performance degradation due to weakened linear features in such complex scenes. However, DeepCalib maintains the lowest errors in these challenging environments, demonstrating its capacity to capture geometric features of curved roads to some extent.

**Figure 12:**
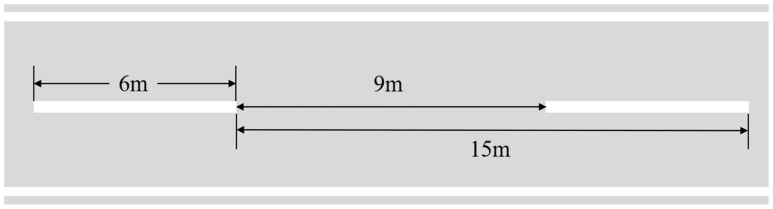
Schematic diagram of three different measurement line segments on the lane.

Based on the visualized experimental setup (Figure 12), we conducted line segment measurements at three distances (6 m, 9 m, 15 m) along standardized highway lane markings. The reference benchmarks (6 m marking intervals and 9 m lane spacing) are explicitly annotated through short line segments and their combinations in the image. Critical measurement lines aligned with lane edges converging toward the vanishing point, enabling direct analysis of perspective projection effects. This experiment compared DeepCalib with manual calibration, traditional methods (AutoCalib and Edgelets), and a deep learning approach (DeepCN). Manual calibration utilized the VWL algorithm from the work [36], where V denotes vanishing point, W represents road width, and L signifies landmark length.

Table 5 presents quantitative results where DeepCalib achieves mean measurements of 6.56 m, 9.96 m, and 16.68 m for the 6 m, 9 m, and 15 m segments, with calibration accuracies of 90.67%, 89.33%, and 88.80%. The overall calibration accuracy reached 89.60%, surpassing AutoCalib (81.46%), Edgelets (76.29%), and DeepCN (86.05%). In contrast, manual calibration achieves centimeter-level precision (≤6 cm). Notably, DeepCalib eliminates scene- and object-specific constraints, demonstrating superior adaptability. This enables high calibration accuracy while maintaining operational flexibility, achieving an optimal balance between precision and generality.

**Figure 13:**
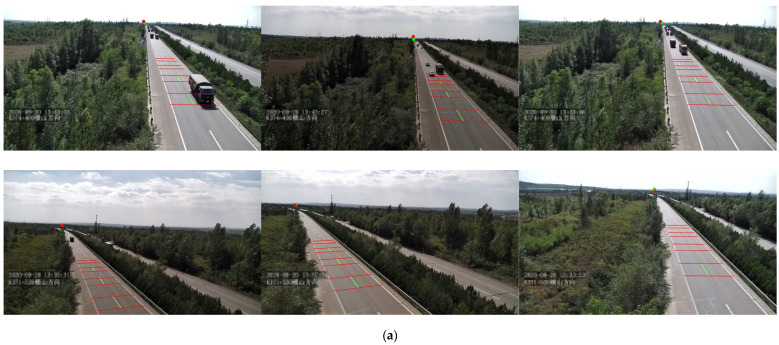

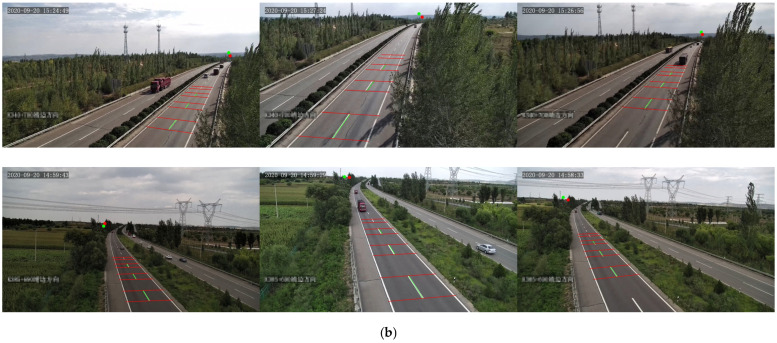
Calibration results of the DeepCalib model in real-world scenarios. (**a**) Straight road environment; (**b**) Curved road environment. Green line segments represent reprojections of the predicted landmark lengths, and red line segments correspond to projections of the lane width.

Figure 13 visually demonstrates the calibration performance of DeepCalib under various surveillance camera perspectives. Green circles mark the ground truth of vanishing points, while red circles indicate predictions. To qualitatively evaluate camera parameter prediction accuracy, we employed the line segment reprojection visualization strategy: green line segments represent reprojections of the predicted landmark lengths, and red line segments correspond to projections of the single lane width (3.75 m). Experimental results indicate that DeepCalib exhibits excellent adaptability to camera perspective variations. Predicted vanishing points show high consistency with ground truth, and the line segment reprojections strictly adhere to perspective transformation principles. Notably, while the errors of vanishing point localization accuracy and projection experience slight increases in curved road scenarios compared to straight sections, overall errors remain within tolerance thresholds. Although current calibration precision still lags behind manual methods, DeepCalib’s advantages lie in its computational efficiency and environmental adaptability. These characteristics make it particularly suitable for dynamic calibration of highway surveillance cameras, offering a practical and scalable solution for intelligent transportation systems.

### 4.5. Time Consumption

To validate the real-time performance of the DeepCalib model in processing 1920 × 1080 resolution image frames, the experimental protocol decomposed the algorithm workflow into two core modules: vanishing point decoding (VP Decoding) and extrinsic parameter estimation (EP Estimation). Comparative methods included traditional multi-stage calibration techniques (AutoCalib [19] and Edgelets [43]) and single-image calibration approach (DeepCN [3]). Table 6 systematically records the time consumption results across processing stages for various calibration algorithms. Comparative analysis reveals that traditional multi-stage methods require tens of seconds for calibration (at 25 FPS video streams), while single-image techniques reduce processing time to the 10^−2^ s range, significantly enhancing real-time efficiency. Notably, computational bottlenecks in traditional approaches like AutoCalib and Edgelets concentrate in the vanishing point estimation phase. This stage requires continuous vehicle tracking and horizontal edge feature extraction to achieve stable vanishing point localization, resulting in processing duration that strongly correlates with traffic volume. Overall, traditional multi-stage methods exhibit significant environmental dependency in processing efficiency, whereas single-image techniques completely circumvent these limitations. Although DeepCalib demonstrates a 3.56 × 10^−2^ s increase in total processing time compared to DeepCN, it still achieves 10 FPS performance. Given its better calibration accuracy, DeepCalib maintains competitive advantages by balancing precision and computational efficiency.

## 5. Conclusions

This study addresses the bottlenecks in existing automatic calibration methods for traffic surveillance cameras, focusing on resolving two critical challenges: the scarcity of labeled datasets and poor adaptability to multi-view scenes. We first constructed a large-scale synthetic dataset through simulation of highway scenarios, establishing an effective data augmentation framework. The synthetic dataset maintains statistical consistency with real-world scenes in terms of geometric feature distribution. By adopting data augmentation techniques such as random color jittering and noise injection, we have alleviated distribution bias in RGB color channels and texture distributions between the two datasets, thereby establishing a solid foundation for transfer learning. Subsequently, we proposed DeepCalib, a deep calibration network that integrates the triplet attention mechanism to enhance the representational capacity of geometric visual cues, enabling simultaneous vanishing point detection and camera extrinsic parameter estimation. The method operates on single highway images without requiring continuous object detection, significantly improving calibration efficiency. To enhance real-world robustness, we adopted the pre-training and fine-tuning strategies. Experimental results on proposed benchmark dataset demonstrate that DeepCalib adapts to diverse highway surveillance camera views. Its simple yet efficient architecture shows practical value for real-world applications. While achieving promising performance, calibration accuracy for curved road scenarios requires further improvement. Future work will focus on expanding the real-world highway dataset to better meet deep learning requirements. Additionally, we aim to strengthen utilization of local visual cues (e.g., vehicles) for more comprehensive perspective feature representation. Addressing these challenges will hold significant potential to advance automatic traffic surveillance camera calibration technology.

## Figures and Tables

**Table 1:** Comparison of the real-world and synthetic dataset parameters.

Dataset	Sample Size	ϕ	θ	*h*	Format	Resolution
MVCCD_R [3]	8765	[−18.4°, 0°]	[−29.3°, 29°]	[10.4 m, 13.9 m]	RGB	1920 × 1080
MVCCD_S	336,249	[−28°, 0°]	[−40°, 40°]	[10 m, 14.5 m]	RGB	1920 × 1080

**Table 2:** Comparison of backbone network ablation studies.

Backbone	L2 Dis (Pixel)	Pitch (°)	Yaw (°)	h (m)
ConvNeXt_tiny	57.15	6.70	10.49	0.98
ConvNeXt_tiny_TAM	**34.69**	**4.43**	**7.84**	**0.98**
ConvNeXt_small	45.98	2.73	5.62	0.94
ConvNeXt_small_TAM	**29.66**	**2.32**	**3.88**	**0.93**
ConvNeXt_base	40.26	1.74	3.94	0.91
ConvNeXt_base_TAM	**22.22**	**1.33**	**2.45**	**0.89**

**Table 3:** Quantitative evaluation of binning strategies on rotation angle estimation.

Angles	Bins Num	Class Accuracy	Residual Error (°)
pitch	2	94.23%	2.78
	**3**	**92.62%**	**1.82**
	4	88.68%	1.49
	5	85.77%	2.97
	6	79.03%	3.35
yaw	2	92.37%	5.27
	3	90.08%	4.84
	4	87.52%	4.05
	**5**	**86.04%**	**2.33**
	6	76.91%	4.45

**Table 4:** Comparison of vanishing point detection performance on different datasets.

Methods	Metrics	MVCCD_R		Dataset [27]
		Straight Roads	Curved Roads	—
AutoCalib [19]	L2 Dis	49.23	77.61	—
	NormDis	0.022	0.035	—
Edgelets [43]	L2 Dis	70.96	130.38	—
	NormDis	0.032	0.059	—
DeepVP [27]	L2 Dis	91.08	119.43	14.64
	NormDis	0.041	0.054	0.035
DeepCN [3]	L2 Dis	57.14	73.16	9.39
	NormDis	0.026	0.033	0.022
DeepCalib	L2 Dis	**13.11**	**34.29**	**7.15**
	NormDis	**0.006**	**0.022**	**0.014**

**Table 5:** Line segment measurement comparison.

Methods	6 m		9 m		15 m		Mean Precision
	Mean Value	Mean Precision	Mean Value	Mean Precision	Mean Value	Mean Precision	
VWL [36]	**6.05**	**99.17%**	**8.91**	**99.00%**	**14.96**	**99.73%**	**99.30%**
AutoCalib [19]	6.95	84.17%	10.92	78.67%	17.77	81.53%	81.46%
Edgelets [43]	7.11	81.50%	11.45	72.78%	18.81	74.60%	76.29%
DeepCN [3]	6.82	86.33%	10.27	85.89%	17.11	85.93%	86.05%
DeepCalib	6.56	90.67%	9.96	89.33%	16.68	88.80%	89.60%

**Table 6:** Processing time comparison between multi-stage and single-image calibration methods.

Methods	VP Decoding (s)	EP Estimation (s)	Total Time (s)
AutoCalib [19]	1.38 × 10^2^	—	1.38 × 10^2^
Edgelets [43]	4.32 × 10^1^	—	4.32 × 10^1^
DeepCN [3]	**6.16 × 10** **^−^** **^2^**	**1.73 × 10** **^−^** **^4^**	**6.18 × 10** **^−^** **^2^**
DeepCalib	9.72 × 10^−2^	1.90 × 10^−4^	9.74 × 10^−2^

## Data Availability

The dataset used in this research has been published in https://github.com/WenTao10/Multi-View-Camera-Calibration-Dataset, accessed on 25 January 2022.

## Abbreviations

PnP	Perspective n point
PTZ	Pan tilt zoom
CNNs	Convolutional neural networks
TAM	Triplet attention module
MVCCD	Multi-view camera calibration dataset
FOV	Field of view
CH	Channel height
CW	Channel Width
VP	Vanishing point
EP	Extrinsic parameter
